# Comparison of Lidocaine and Mepivacaine for Variation in Regional Tissue Oxygenation in Stellate Ganglion Block: A Randomized, Double-Blind, Crossover Trial

**DOI:** 10.7759/cureus.47938

**Published:** 2023-10-30

**Authors:** Hidetaka Kuroda, Norika Katagiri, Keita Kagawa, Uno Imaizumi, Noboru Ishikawa, Yoshiyuki Shibukawa, Yoshinari Morimoto, Takuro Sanuki

**Affiliations:** 1 Department of Dental Anesthesiology, Kanagawa Dental University, Yokosuka, JPN; 2 Department of Forensic Odontology and Anthropology, Tokyo Dental College, Chiyoda, JPN; 3 Department of Physiology, Tokyo Dental College, Chiyoda, JPN; 4 Department of Geriatric Dentistry, Kanagawa Dental University, Yokosuka, JPN

**Keywords:** stellate ganglion block, regional tissue oxygenation, regional blood flow, mepivacaine, lidocaine

## Abstract

Introduction

This prospective, randomized, double-blind, crossover trial aimed to investigate the effect of different types of local anesthetics on regional tissue blood oxygenation on the stellate ganglion block (SGB).

Methods

Twenty eligible patients were recruited for this study; 16 of whom were allocated to the study protocol. Participants were randomized into one of the two crossover sequences: 1% lidocaine hydrochloride or 1% mepivacaine hydrochloride, and received SGBs with 6 mL of any one of the local anesthetics, followed by a washout period of more than 24 hours, and then received SGBs by substituting the two local anesthetics. The tissue oxygenation index (TOI) in the skin near the mental foramen on the blocked side was recorded using near-infrared spectroscopy at 15 minutes after the local anesthetic injection.

Results

One participant did not complete the study. As such, variation in regional tissue oxygenation was compared between the 15 participants. There was no difference in the increase in regional tissue blood flow or tissue oxygenation after SGB between the patients who were given lidocaine and the ones who were given mepivacaine; however, the kinetics of the increase in regional tissue oxygenation were significantly faster with mepivacaine than with lidocaine.

Conclusions

Different types of local anesthetics do not affect the intensity of the increase in regional tissue oxygenation after SGB, but they do affect the kinetics of the increase. These differences in local anesthetics may affect how patients feel after receiving SGB, the duration of SGB effects, and the frequency of adverse events associated with SGB.

## Introduction

The stellate ganglion block (SGB) is an interventional treatment aimed at improving painful conditions such as complex regional pain syndrome and postherpetic neuralgia in the orofacial region as well as blood flow disorders such as upper extremity ischemia [1-4]. Some of these orofacial pains are maintained via sympathetic nerve activity, and the SGB, which is a cervical sympathetic nerve block, can show therapeutic efficacy. The SGB is injected with a local anesthetic as a landmark with the sixth or seventh cervical vertebra to transiently block cervical sympathetic chain activity. When injected into the neck, numerous possible complications are associated with the SGB, including recurrent laryngeal nerve and brachial plexus palsy and local anesthetic systemic toxicity [3,5,6]. Therefore, to minimize damage due to complications, the Japan Society of Pain Clinicians recommends short-acting 0.5-1.0% lidocaine hydrochloride (lidocaine) or mepivacaine hydrochloride (mepivacaine) as local anesthetics for the SGB [7].

For the treatment of paralytic diseases, such as facial nerve palsy and trigeminal nerve palsy, the SGB is performed with the expectation of increasing regional tissue blood flow and oxygenation [8,9]. In our previous study, we found that unilateral SGB using 1% lidocaine resulted in an increase in tissue oxygenation in the mandibular region and resulted in the recovery of postoperative trigeminal nerve palsy [10]. Lidocaine and mepivacaine have different pharmacological effects on blood vessels; lidocaine has a vasodilation effect, whilst mepivacaine has a vasoconstriction effect [11,12]. The compartment injected with the local anesthetic in the SGB includes the carotid artery, which is the main trunk of the artery innervating the head and the face, where the SGB is required to be effective. Changes in the diameter of the carotid artery due to the local anesthetic used for the SGB may affect blood flow and oxygenation in the periphery. Moreover, the selection of the local anesthetic could impact the treatment's efficacy for facial nerve and trigeminal nerve palsy. Therefore, this study hypothesized that different types of local anesthetics affect regional tissue blood flow and oxygenation differently. To test our hypothesis, we aimed to compare the variations in regional tissue oxygenation following lidocaine or mepivacaine SGBs using a near-infrared oxygenation monitor.

This article was previously posted to the Research Square preprint server on November 23, 2022.

## Materials and methods

Study participants and settings and locations

Twenty patients with postoperative trigeminal neuropathy, referred to the Department of Anesthesiology and Pain Relief Center, Kanagawa Dental University Hospital, Yokosuka, Japan, for treatment were screened between May 2020 and March 2021. The eligibility criteria were a diagnosis of unilateral trigeminal disturbance with a history of nerve injury due to dental surgery and age ranging from 17 to 80 years old. The diagnosis was made by a dental anesthesiologist specializing in pain. All patients were new to SGB. Exclusion criteria were contraindications or decline to SGB, American Society of Anesthesiologists physical status >3, or no consent provided by the participant. This study was approved by the Ethics Committee of Kanagawa Dental University (approval number: 597), conformed to the provisions of the Declaration of Helsinki, and was registered with the UMIN (University Hospital Medical Information Network) Clinical Trials Registry (UMIN000040404, dated May 15, 2020). Written informed consent was obtained from all participants.

Study design and intervention

**Figure 1:**
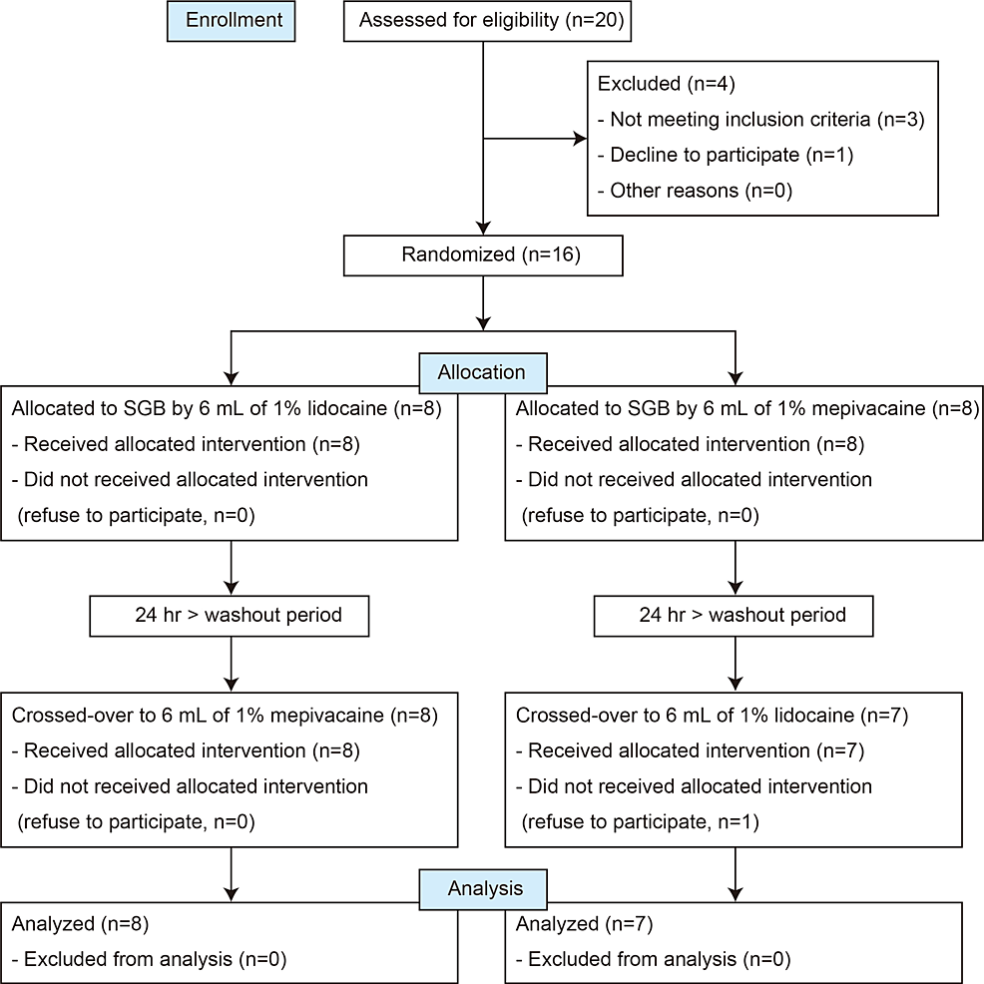
CONSORT flow diagram showing the number of participants through each stage of the randomized crossover trial. The tissue oxygenation index was assessed during the SGB. CONSORT: Consolidated Standards of Reporting Trials; SGB: stellate ganglion block

We designed and implemented a double-blind crossover study to investigate changes in mandibular tissue oxygenation on the blocked sides during SGB using two different local anesthetics. The crossover study timeline is shown in Figure 1.

Before the initial SGB, participants were randomly and equally allocated to the two protocols using sequentially numbered envelopes. Each sealed envelope contained an allocation note. A study participant randomly selected one envelope from the collection. The person in charge of the allocation and assignment opened the envelope and prepared the local anesthetic of the assigned group. The two protocols were SGB with 6 mL of 1% lidocaine (Aspen Japan, Tokyo, Japan) or SGB with 6 mL of 1% mepivacaine (Sandoz Pharma, Tokyo, Japan). As described previously [10], all unilateral SGB procedures were performed using a paratracheal approach by the same operator. Successful SGB was confirmed by the presence of Horner's syndrome (i.e., drooping eyelids, constricted pupils, enophthalmos, and stuffy nose) at the blocked side. After a washout period of >24 hours, SGB was performed by substituting the two local anesthetics. The person in charge of the allocation and assignment was different from the SGB operator. The former prepared the local anesthetic and the latter was blinded to the local anesthetic used. In addition, the outcome assessors differed and participants were not told which local anesthetic they had been injected with, resulting in a double-blind design. Considering the durations of action of both local anesthetics [13,14], a carryover effect is ruled out because the durations of action of lidocaine and mepivacaine are short. The study was terminated at a patient's request or when complications due to the SGB occurred.

Outcome measurement

The primary outcome was tissue oxygenation at 15 minutes after local anesthetic injection to the skin near the mental foramen on the blocked side. In our previous report, tissue oxygenation after SGB was maximal at 15 minutes [10]. The tissue oxygenation index (TOI) was recorded using near-infrared spectroscopy (NIRS) performed using a near-infrared oxygenation monitor (NIRO-200NX; Hamamatsu Photonics, Shizuoka, Japan). The NIRO-200NX could measure TOI without being influenced by factors, such as hemoglobin concentration, transcellular fluid volume, and bone thickness, that typically affect the optical path length [15]. The TOI was obtained using the following equation: TOI (%) = oxygenated hemoglobin concentration (O_2_Hb) / O_2_Hb + deoxygenated hemoglobin concentration and was automatically calculated using the NIRO-200NX monitor software. The variation in TOI (*Δ*TOI) was expressed as the change from the value at the end of the local anesthetic injection.

Statistical methods

Data are expressed as mean ± 95% confidence interval (CI). A paired *t*-test was used to determine parametric statistical significance. Statistical significance was set at *p* < 0.05. All statistical analyses were performed using GraphPad Prism version 7.05 statistical software package (Dotmatics, Boston, Massachusetts, United States). The kinetics of the increase in TOI after SGB was determined by fitting the experimental data with a single exponential function using OriginPro 2020 software (OriginLab Corporation, Northampton, Massachusetts, United States). Additional subgroup analysis was performed to evaluate the effects of variables (age, sex, body mass index, and affected side) on the differential effects of lidocaine and mepivacaine.

Sample size estimation was performed using data obtained from our previous reports [10]. The variation in TOI between the two different groups 15 minutes after SGB showed a difference of 3.99%, with a standard deviation (SD) set at 2.98%. To detect a difference in TOI variation between the two groups using a two-tailed paired *t*-test with a type I error of 0.05 and a power of 0.8, nine subjects were required. To account for the possibility of participant withdrawals from the study, a total of 20 patients were included. Considering the possibility of dropping out of the protocol, 20 participants were required.

To assess whether the sample size was adequate, post-hoc power analyses were used to determine the statistical power of the *t*-test using the G*Power version 3.1.9.6 statistical software package G*Power version 3.1.9.6 (2020; Heinrich-Heine-Universität Düsseldorf, Düsseldorf, Germany).

## Results

Twenty eligible patients were recruited for this study (Figure 1). One patient declined to participate and three patients were excluded because they did not meet the inclusion criteria. The four patients were treated with pharmacotherapy alone. The remaining 16 participants were randomized to one of the two study protocols (Figure 1). The dental surgeries that led to nerve injury included jaw deformity surgery in seven cases, lower wisdom tooth extraction in seven cases, and lower molar root canal treatment in two cases. There was a protocol deviation, in which one patient had a headache after the initial SGB and refused to participate in the second intervention. Outcomes were analyzed for the 15 participants for whom data for both local anesthetics were available (Figure 1). The demographics and characteristics of the participants are presented in Table 1.

**Table 1:** Demographics and characteristics Data are expressed as the mean ± 95% confidence interval or number of subjects. BMI: body mass index

Patient Characteristics	
Age (year)	37.93 ± 7.48
Gender (female/male)	10 / 5
Height (cm)	163.09 ± 3.80
Weight (kg)	59.84 ± 5.62
BMI (kg/m^2^)	22.43 ± 1.71
Affected side (right/left)	7 / 8

Increase in *Δ*TOI between lidocaine and mepivacaine after SGB

**Figure 2:**
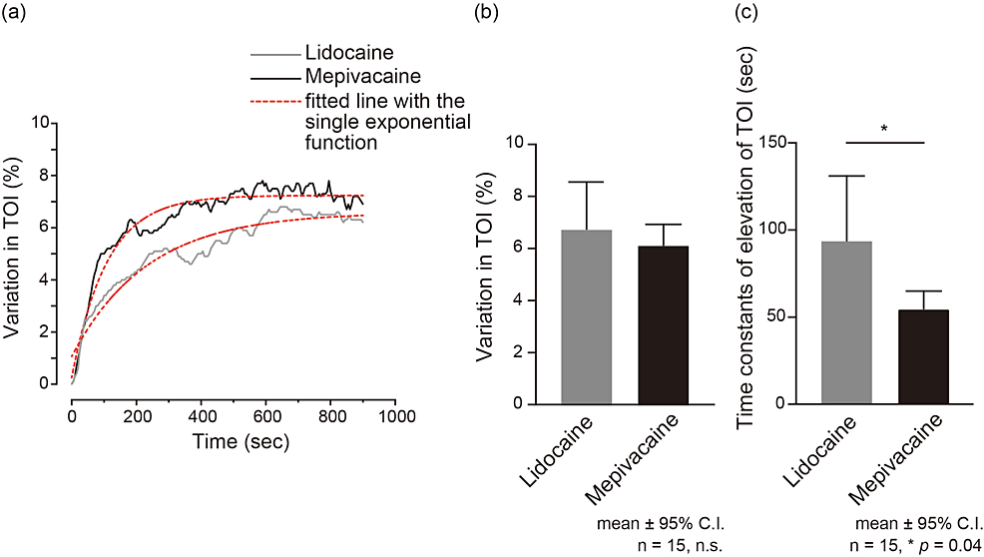
Changes in the variation in tissue oxygenation after stellate ganglion block (SGB) (a) Representative trace of an increase in the TOI. The values at the end of the injection of SGB (0 minutes) were used as the baseline. The TOI increased logarithmically by SGB using lidocaine (grey line) and mepivacaine (black line). The value of the TOI represents the percentage of O_2_Hb contained in the tissue and was calculated by fitting with a single exponential function. Changes in the variation in TOI were well fitted by a single exponential function (red dotted line). The variation in TOI by SGB using mepivacaine showed a rapid increase, while that of lidocaine exhibited a slower rate of increase in kinetics. (b) Summary bar graphs of the increase in variation of TOI after SGB using lidocaine (grey column) and mepivacaine (black column). (c) Summary bar graphs of time constants of increase in TOI after SGB using lidocaine (grey column) and mepivacaine (black column). Each bar denotes the mean (± 95% confidence interval) of all subjects. Statistically significant differences between columns are indicated by an asterisk: *p < .05 based on a paired* t*-test. n.s.: not significant; TOI: tissue oxygenation index; O2Hb: concentration of oxyhemoglobin; sec: seconds

When the SGB was performed, *Δ*TOI showed an exponential increase for both local anesthetics (Figure 2a). The increase in *Δ*TOI after 15 minutes of SGB was 6.75% (95% CI: 4.95-5.55) for lidocaine and 6.14% (95% CI: 5.35-6.93) for mepivacaine (Figure 2b). No statistically significant differences were observed in the *Δ*TOI increase between the two local anesthetics (*p*=0.47) (Figure 2b). By fitting to a single exponential function (red dotted line in Figure 2a), the increasing response of *Δ*TOI was obtained using the time constants. The ΔTOI following SGB with mepivacaine demonstrated a swift increase (54.96 sec, 95% CI: 44.97-64.95), whereas lidocaine exhibited a slower rate of increase in kinetics (92.08 sec, 95% CI: 54.04-130.1) (p=0.04) (Figure 2c). Post-hoc power analyses showed the achieved powers (1 - β err prob) for the TOI and time constant between lidocaine and mepivacaine were 0.12 and 0.58, respectively.

Varying with variables by patient characteristics

In ancillary subgroup analysis, we examined whether the difference in efficacy between lidocaine and mepivacaine varied based on patient characteristics. The subgroups were divided based on the median patient characteristics or the affected side. When the difference in efficacy between lidocaine and mepivacaine was compared within each subgroup, the primary outcome was consistent with the results in each subgroup (Table 2).

**Table 2:** Variation in TOI and time constant according to age, sex, BMI, and affected side Data are expressed as mean ± 95% confidence interval (number of subjects). Numbers in parentheses indicate the number of participants. Subgroups were created based on the median values for numbers that could be indicated by continuous variables. Each variable had no effect on TOI or the time constant of TOI elevation. TOI: tissue oxygenation index; BMI: body mass index; sec: seconds

	Variation in TOI (%)		
	Lidocaine	Mepivacaine	P-value
Age (year)			
≤ 38	5.66 ± 2.46 (8)	6.35 ± 1.34 (8)	0.58
> 38	8.00 ± 3.10 (7)	5.90 ± 1.17 (7)	0.07
BMI (kg/m^2^)			
≤ 21.6	6.71 ± 3.09 (8)	6.55 ± 1.07 (8)	0.91
> 21.6	6.80 ± 2.74 (7)	5.671 ± 1.41 (7)	0.26
Gender			
Female	6.80 ± 2.28 (10)	6.23 ± 0.89 (10)	0.58
Male	6.66 ± 4.68 (5)	5.96 ± 2.33 (5)	0.7
Affected side			
Right	6.73 ± 2.40 (7)	6.27 ± 1.26 (7)	0.67
Left	6.78 ± 3.29 (8)	6.03 ± 1.31 (8)	0.6
	Time constant of elevation of TOI (sec)		
Age (year)			
≤ 38	98.14 ± 67.36 (8)	49.03 ± 11.63 (8)	0.11
> 38	85.15 ± 53.39 (7)	61.73 ± 19.25 (7)	0.2611
BMI (kg/m^2^)			
≤ 21.6	88.42 ± 55.40 (8)	46.85 ± 13.98 (8)	0.12
> 21.6	96.25 ± 70.67 (7)	64.23 ± 14.48 (7)	0.25
Gender			
Female	81.25 ± 42.15 (10)	53.3 ± 12.32 (10)	0.13
Male	113.70 ± 109.80 (5)	58.27 ± 29.37 (5)	0.23
Affected side			
Right	92.18 ± 63.09 (7)	46.63 ± 15.74 (7)	0.11
Left	91.99 ± 61.51 (8)	62.24 ± 13.81 (8)	0.26

## Discussion

Our results demonstrate no difference in the increase in regional tissue blood flow or tissue oxygenation after lidocaine or mepivacaine SGB; however, the kinetics of the increase in regional tissue oxygenation were faster with mepivacaine than with lidocaine.

This study is the first to explain the effects of different local anesthetics on the SGB, although there are numerous reports that the SGB accelerates regional tissue oxygenation [1,10,16-23]. The lack of difference in regional oxygenation capacity provides a positive interpretation of previously reported therapeutic benefits of the SGB. In this study, differences in the kinetics of the increase in regional tissue oxygenation were observed with different local anesthetics. Although there was no difference in clinical efficacy (i.e., increased regional tissue oxygenation and blood flow), the kinetics of the effect were different, and the difference in the local anesthetics may have led to differences in the occurrence of Horner's syndrome and the patient's feeling of warmth. Moreover, these results indicate that the local anesthetic injected with the SGB induces vasoconstriction or vasodilation in blood vessels placed in the same compartment. Mepivacaine causes a vasodilation effect, while lidocaine causes a vasoconstriction effect [11,12]. When the cardiac output and the vascular resistance remained constant, a smaller vessel diameter would generally increase the blood flow velocity. As a result, the increase in tissue oxygenation may have accelerated more with mepivacaine as compared to lidocaine. The onset of mepivacaine and lidocaine was reportedly approximately eight minutes, and the elimination half-life of those anesthetics was 100 minutes [24,25]. Given that both of the pharmacokinetics were comparable, it is reasonable to assume that the outcome in this study was influenced by the effects of each local anesthetic on the blood vessels. The SGB has a number of possible complications, some of which may be influenced by the kinetics of rising serum local anesthetic concentrations [26]. Rapid increases in blood concentrations of local anesthetic can lead to severe local anesthetic toxicity. Although a gradual acceleration of tissue blood flow may be a safer approach for SGB, further investigation is needed to determine the frequency of complications due to the SGB using different types of local anesthetics. In addition, the differences in vascular effects of the two distinct local anesthetics in this study could potentially impact the duration of SGB effects. Additional investigation is also required to further explore the duration of the SGB effects.

This study had several limitations. First, the post-hoc power analyses in this study gave results ranging from small (0.12) to medium (0.58) suggesting the number of participants was small, although the sample size was calculated based on our previous report [10]. However, given the homogeneity in diseases of selected participants, the relative variability in the characteristics of the study participants, and the general pharmacological characteristics of lidocaine and mepivacaine for blood vessels [11,12], we could explain the generalizability of this study. Second, although the increase in tissue oxygenation was not significant between the local anesthetics, the middle- to long-term patient outcomes were not clarified. Further large-scale follow-up studies are necessary to answer this question. This study showed an objective outcome with a short data-acquisition period. Stated differently, this protocol did not include seasonal effects and/or effects of the order of intervention, which depended on the subjectivity of the participants. Since the duration of action of lidocaine and mepivacaine is approximately two to three hours [13,14], potential carryover effects were not considered in this study.

## Conclusions

This study examined the differences in oxygenation between lidocaine and mepivacaine in SGB. In conclusion, different types of local anesthetics do not affect the intensity of the increase in regional tissue oxygenation after SGB, but they do affect the kinetics of the increase. Although it is difficult to conclude which local anesthetic is more suitable for SGB in this study, differences in local anesthetics on SGB may impact patients' postoperative experiences, the duration of SGB effects, and the occurrence of adverse events associated with SGB.
